# I thought I saw a pussy cat: Portrayal of wild cats in friendly interactions with humans distorts perceptions and encourages interactions with wild cat species

**DOI:** 10.1371/journal.pone.0215211

**Published:** 2019-05-01

**Authors:** Esther van der Meer, Sandra Botman, Simone Eckhardt

**Affiliations:** 1 Cheetah Conservation Project Zimbabwe, Victoria Falls, Zimbabwe; 2 Saxion, Department of Tourism Management, Deventer, the Netherlands; 3 Stichting SPOTS, Stampersgat, the Netherlands; Ashoka Trust for Research in Ecology and the Environment, INDIA

## Abstract

Most people lack the opportunity to see non-domesticated animals in the wild. Consequently, people’s perception of wild animals is based on what they see on (social) media. The way in which (social) media portrays non-domesticated animals determines our perception of and behaviour to these animals. People like to interact with animals, which is why venues which offer the opportunity to interact with non-domesticated animals are popular wildlife tourist attractions (WTAs). However, these WTAs more often than not profit at the expense of animal welfare, conservation and human safety. Participation in such WTAs should therefore be discouraged. Through (social) media we are regularly exposed to images of non-domesticated animals in close interactions with humans. Exposure to such images seems to blur the line between what is a friendly domesticated animal and what is a potentially dangerous wild animal. Such images may also increase our desire to engage in interactions with non-domesticated animals ourselves and reduce moral concerns about the use of non-domesticated animals for such interactions, thereby promoting WTAs in which tourists can interact with non-domesticated animals. Wild cat species are commonly used in the wildlife tourism industry to interact with tourists. In this study, we determine whether portrayal of wild cat species in interactions with humans promotes WTAs with wild cats. We presented respondents with an image of a wild cat species (lion, cheetah, caracal) in a control setting, walked by a human (WTA), petted by a human (WTA) or in the wild and asked them to answer a fixed set of questions. We found that portraying wild cat species in interactions with humans reduced the fear of wild cats, encouraged people to regard WTAs with wild cats as acceptable and stimulated them to participate in such activities themselves.

## Introduction

Humans have a desire to view and interact with (non-domesticated) animals [1,2]. Connecting with animals has been shown to evoke positive emotions and improve human health and well-being [2,3]. Simply observing an animal can already reduce stress and increase positive mood [4]. It is therefore not surprising that people experience friendly encounters in which they can get close to, touch, and feel a connection with animals as very satisfying and emotional [5,6]. Our desire to interact with (non-domesticated) animals is so strong we are willing to pay for such encounters [7–9] and interactions with non-domesticated animals are popular wildlife tourist attractions (WTAs) [9]. Millions of animals [10] annually provide entertainment for at least 100 million tourists worldwide [11], generating substantial economic benefits for the tourist destinations involved [9]. Excluding hunting and fishing, WTAs are based on non-consumptive encounters with non-domesticated animals in the wild or in captivity [12] and cover a wide range of activities varying from excursions to watch free-ranging species like whales [13] or turtles [14] to direct interactions [9] like ride elephants [8] or pet and/or walk with lions [7].

WTAs can make a positive contribution to conservation by raising awareness, promoting pro-conservation attitudes and behaviour and providing socio-economic incentives for the long-term conservation of wildlife and their natural habitats [15,16]. However, a study by Moorhouse et al [9] showed most WTAs have negative consequences for conservation and/or animal welfare. The presence and behaviour of unfamiliar people can be stressful for non-domesticated animals, resulting in elevated levels of stress hormones and increased agonistic and atypical behaviour [17–19]. WTAs which provide the opportunity to directly interact with wildlife are particularly likely to cause stress, as non-domesticated animals are forced to be in close proximity to humans with limited control over whether or not they interact with those humans [20,21]. In addition, non-domesticated animals used to interact with tourists often suffer from additional stressors which further impede their welfare, such as being separated from their mother at a very young age and/or beaten and harmed to be trained to interact with humans [7,22,23]. The negative implications of WTAs on animal welfare and conservation have been the subject of animal welfare organisations’ awareness campaigns [22,23]. This has in some cases resulted in the adoption of animal welfare guidelines in the tourism branch [24,25]. Nevertheless, the majority of the tourists participating in WTAs seem unaware of the potentially dire consequences of these attractions and leave positive reviews on online platforms for activities which have a clear negative impact on animal welfare and conservation [9].

Few people have the opportunity to learn about wildlife through first-hand experience in the wild, the majority of us establish a relationship with wildlife through (social) media [26,27]. Exposure to (social) media affects behaviour [28,29] and shapes beliefs, attitudes and social norms [30–32]. For example, watching classic wildlife documentaries has been shown to promote pro-conservation attitudes and behaviour [33,34]. The way in which non-domesticated animals are portrayed in (social) media determines how we perceive them, e.g. the danger they represent, their suitability as pets and whether they are endangered in the wild [35–39]. Consequently, inappropriate portrayal of non-domesticated animals distorts our perceptions of wildlife and creates misconceptions and false expectations [26,37–40].

In recent years, new technologies, the introduction of animal dedicated cable television networks and a changing viewer demand have led to the development of wildlife series which, in contrast to traditional wildlife documentaries, try to draw viewers by showing direct interactions between humans and non-domesticated animals [27,40,41]. We not only get exposed to images of close interactions between humans and non-domesticated animals through these popular wildlife series (e.g. [42]), such interactions are also relatively common subjects of regular television programmes(e.g. [43]), commercials (e.g. [44,45]), fashion shoots (e.g. [46,47]) and social media posts by celebrities (e.g. [48,49]). Exposure to images of non-domesticated animals in close interactions with humans seems to blur our perception of what is a friendly domesticated animal and what is a potentially dangerous wild animal [26,37,38,50], thereby impeding our ability to accurately judge how our behaviour towards wild animals impacts on animal welfare and conservation [39] and what danger non-domesticated animals represents [26,37,38]. Moreover, watching images of humans interacting with non-domesticated animals through (social) media has the potential to increase our own desire to participate in WTAs which offer the opportunity to engage in such interactions [28,29,51] and reduce our moral objections to these tourist activities [52,53].

In this study, we focus on the portrayal of three wild cat species which are used in the tourism industry to be petted by or to walk with tourists: lion *(Panthera leo)*, cheetah *(Acinonyx jubatus)* and caracal *(Caracal caracal)* (e.g. [54–59]). We presented respondents with an image of one of these wild cat species in a neutral setting (control), petted by a human, walked by a human or in the wild and asked a fixed set of questions to determine if portrayal affected 1) people’s intention to engage in WTAs in which they can interact with wild cat species, 2) people’s perception of the suitability of wild cat species to be used for such WTAs, 3) people’s perception of the danger wild cat species represent. Ross et al [37] showed that portrayal of chimpanzees with humans negatively affects people’s perception of their conservation status and suitability as a pet. In this study, we therefore included questions about the perception of wild cats as suitable pets and their conservation status in the wild. We predicted that respondents presented with an image of a human-wild cat interaction would be more inclined to interact with wild cats, more likely to perceive wild cats as suitable to interact with tourists and less fearful of wild cats. We expected this effect to be strongest for 1) the image in which a wild cat is being petted by a human, 2) the smallest wild cat species.

## Methods

### Interviews

**Fig 1:**
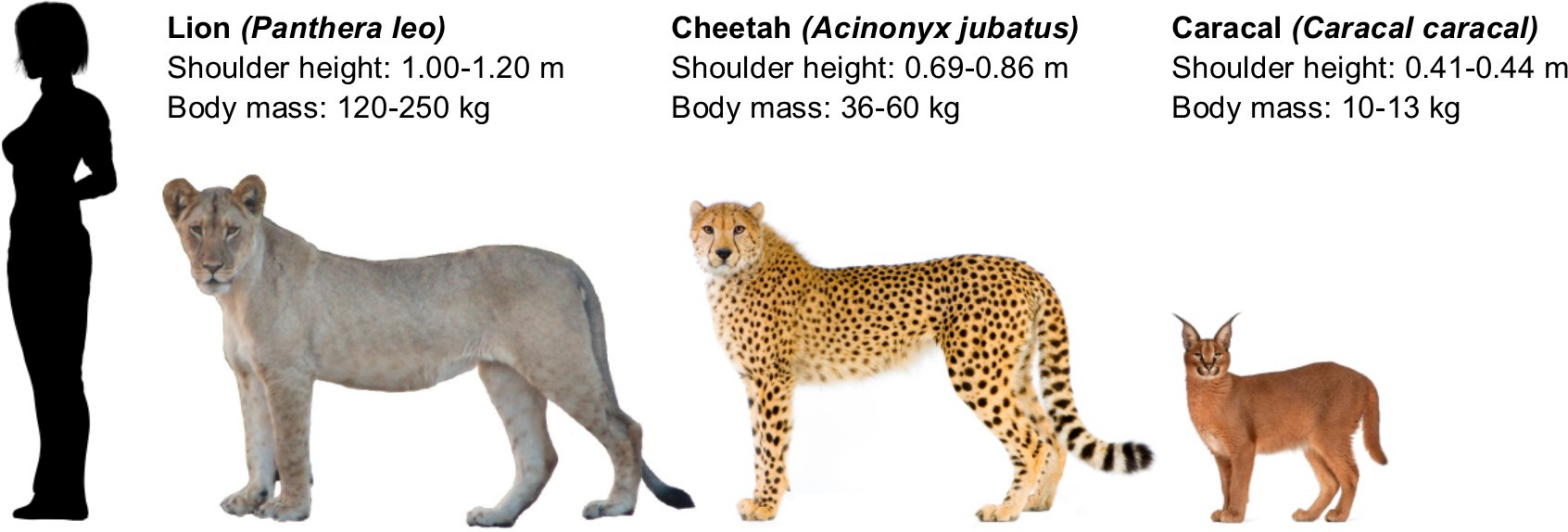
Illustration of the three cat species in this study. Name, scientific name, shoulder height and body weight of caracal, cheetah and lion.

**Fig 2:**
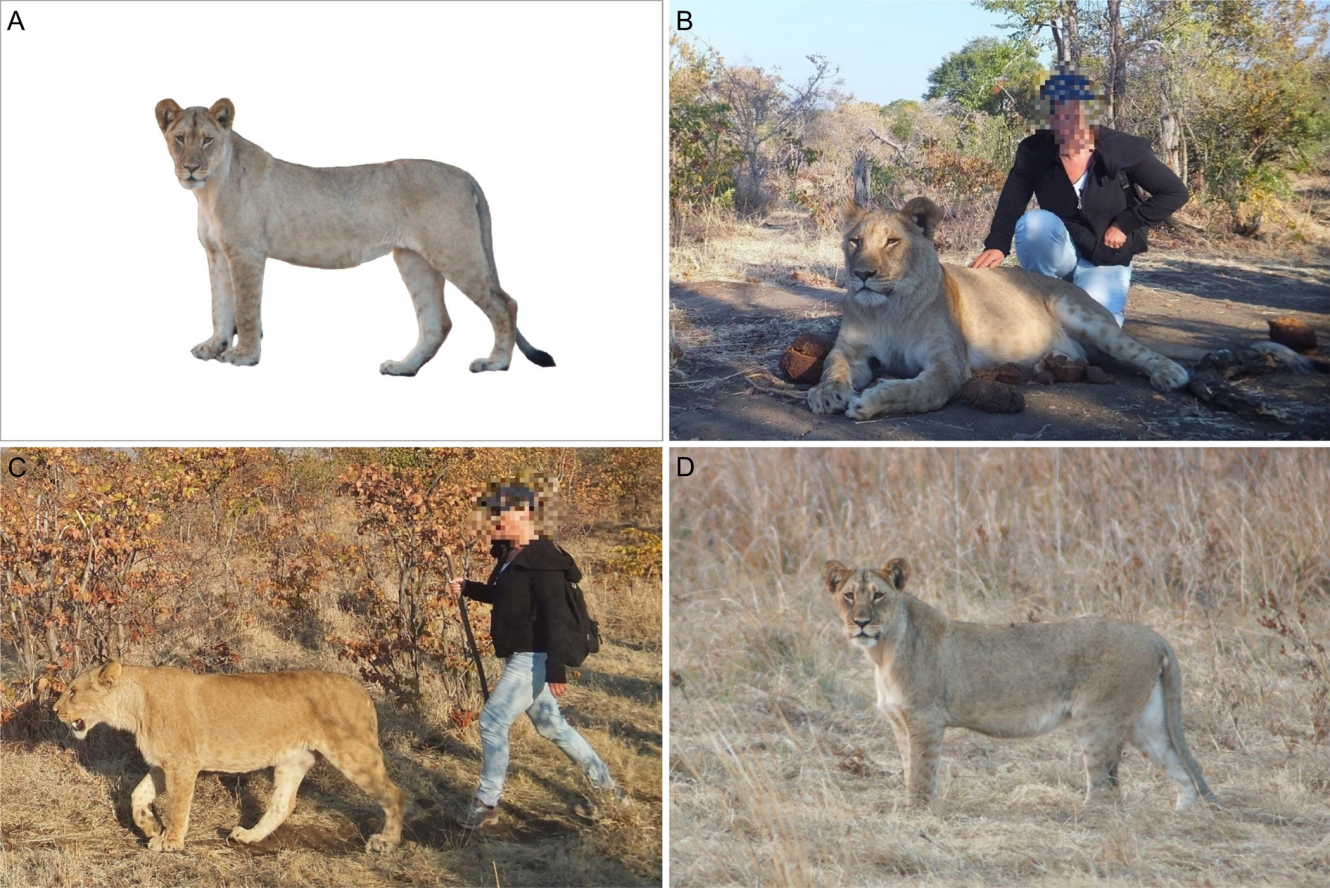
Example of an image set used in the survey. (A) control image of the species against a white background, (B) species being petted by a human, (C) species being walked by a human, (D) species in the wild. Faces have been blurred for publication purposes.

To assess whether portrayal of wild cat species affects people’s intention to engage in interactions with wild cats and their perception of 1) the suitability of wild cats to interact with tourists, 2) the danger wild cats represent, 3) the suitability of wild cats to be kept as pets and 4) the conservation status of wild cats in the wild, we conducted a questionnaire-based survey in which we presented respondents with different images of wild cat species. Perceptions of (non-domesticated) animals can vary considerably per species, even if those species belong to the same taxonomic family [60]. In this study, we used A5 colour images of three different wild cat species (caracal, cheetah, lion) which are all used in WTAs but, due to differences in size and appearance (Fig 1), may elicit a different response from our respondents. These wild cat species were portrayed against a white neutral background (control), petted by a human (human sitting next to the animal with a hand placed on it), walked by a human or in the wild. Both petting and walking wild cats are activities commonly offered as WTAs [7,9,54–57]. An example of an image set can be found in Fig 2. The images were sourced online and represent real situations in the wild or semi-captivity in which tourists interact with wild cats. We made sure, between species, that the images were comparable in composition, that animals and humans had similar postures, that facial expressions and clothing were comparable, that the human who petted or walked the wild cat was a Caucasian female and that references to captivity (e.g. fences) were removed (Fig 2). Where necessary we manipulated images using Photoshop (Adobe Systems, San Jose, California, United States of America).

Between November and December 2017, we approached members of the public at 8 main railway stations in the Netherlands for one-on-one interviews. We interviewed 649 respondents using a structured questionnaire with a mix of open- and closed-ended questions (S1 Appendix). Each question with all its answer options was read out loud after which the respondent chose his/her preferred answer option. Answer options for closed-ended questions were either dichotomous (yes, no), trichotomous (yes, maybe, no) or followed a five-point Likert scale [61] (S1 Appendix). An interview started with questions about demography and background characteristics (S1 Appendix). We subsequently presented the respondent with an image of one of the three wild cat species portrayed in one of the four settings. After we had given the respondent time to view the image, we asked him/her whether (s)he would like to be photographed with, walk with or pet the animal in the image and whether (s)he believed this animal was suitable to be photographed with, walked with or petted by tourists. We also determined what danger the wild cat in the image represents to respondents, whether respondents perceived it as a nice pet and what conservation status respondents believed it had in the wild (S1 Appendix).

Participation in this study was voluntary and fully anonymous. We only interviewed respondents who, prior to starting the interview, gave consent to use the information they provided in this study. To ensure full anonymity, this consent was given verbally. At any time, respondents were free not to answer questions or abort the interview. This research and the above-mentioned procedure to obtain consent was approved by the Ethical Review Committee Psychology and Neuroscience of the University of Maastricht under reference number ERCPN-EXTERN-196-02-07-2018.

### Analyses

In order to secure a sufficient number of events per variable for statistical analyses [62,63], we collapsed the answer options, which followed a five-point Likert scale (S1 Appendix), into three answer categories by combining the categories suitable and very suitable, unsuitable and very unsuitable, sweet and very sweet, dangerous and very dangerous. Because the data represent categories with intrinsic order, we fitted ordinal logistic regression models to assess whether the image shown to the respondent affected the respondents’ intention to engage in interactions with wild cat species and their perception of the suitability of wild cat species to interact with tourists, the danger wild cat species represent, suitability as pets and conservation status in the wild. An ordinal logistic regression model was not appropriate (Pearson’s goodness of fit test p < 0.05, test of parallel lines p < 0.05) to assess whether the image shown affected the respondents’ perception of wild cat species as sweet, neutral or dangerous. Instead we used a multinomial regression model to analyse this dependent variable.

Few respondents perceived wild cat species as nice pets. Therefore, to ensure a sufficient number of events per variable [62,63], it was decided to analyse this dependent variable by using a binary logistic regression model with ‘not a nice pet’ (answer option no) as the reference and ‘might be a nice pet’ (combined answer options yes and maybe) as the response variable. None of the respondents thought wild cat species are very suitable to be kept as pets, only nine respondents believed wild cat species are suitable pets. Due to this small sample size, the number of events per variable were insufficient to perform further statistical analyses [62,63].

Image type (control, petted, walked, wild) and species in the image (caracal, cheetah, lion) were added to the models as a factor. Several studies have shown the demographic variables gender, age and education determine how we view and relate to animals [64–68], we therefore controlled for gender, age (≤ 24, 25–44, 45–64 and ≥ 65) and level of education by adding these variables to each model as factors and covariate respectively. Several of the respondents had participated in WTAs with non-domesticated animals. The type of WTAs respondents had engaged in were categorised into four categories (no previous engagement, direct interactions with animals, visiting captive animal facilities, going on a safari to watch animals) and included in the model as a factor. In a few cases (n = 11) respondents had engaged in direct interactions with non-domesticated animals but had also visited captive facilities or been on a safari to watch non-domesticated animals, these respondents were classified as having had direct interactions with animals. Preliminary analyses revealed the two-way interaction image type x species did not affect intention to interact with wild cats nor did it affect perception of wild cat species as suitable to interact with tourists, the danger wild cat species represent, suitability as pets and conservation status in the wild (0.10 ≤ p ≤ 0.72, 5.17 ≤ χ^2^ ≤ 11.81) and was therefore excluded from the final analyses.

To allow for a comparison between models and prevent potential bias in parameter estimation (β) [62] and therewith odds-ratio (exp β), we decided to present full models (see e.g. [69,70]). Statistical analyses were performed with SPSS software version 20.0 (SPSS Inc., Chicago, IL, U.S.A.), using a GENLIN procedure based on likelihood-ratio tests. Variables with a p-value > 0.05 and/or odds-ratio for which the 95% confidence interval included 1.00 were considered not to affect the dependent variable. In addition, we used Pearson’s χ^2^-square tests to determine whether the intention to interact with wild cats (yes or no), the perception of the suitability of wild cats to interact with tourists (suitable or unsuitable) and the perception of wild cats as pets (might be a nice pet or not a nice pet) was affected by the perceived danger (sweet or dangerous) wild cat species represent.

## Results

### Characteristics respondents

For each wild cat species and type of image combination (12) we interviewed ≥ 48 respondents. Across the twelve images, gender of our respondents was minimum 44.4% and maximum 58.5% female, mean age per image varied from 34.9 ± 2.5 (mean ± SE) to 40.1 ± 2.8 (mean ± SE) and level of education ranged from 5.2 ± 0.3 (mean ± SE) to 5.9 ± 0.3 (mean ± SE) which is the equivalent of higher general secondary education (HAVO in the Dutch educational system) and pre-university education (VWO in the Dutch educational system). Respondents resided in 244 different locations, varying from little villages (rural environment) to large cities (urban environment). The majority of the respondents grew up with animals and/or kept pets (89.8%).

A large percentage of the respondents learned about wildlife by watching series and documentaries on television (77.9%) and/or visiting zoos (72.1%), while some read animal and/or nature related magazines (22.7%). Most respondents watched wildlife series on animal dedicated cable television networks (i.e. Animal Planet, Discovery, National Geographic) (36.6%), Dutch television channels (23.3%) and/or European channels (12.3%). Forty percent of the respondents had participated in ≥ one WTA with non-domesticated animals during their holidays. These WTAs varied from direct interactions (20.7%) to visiting captive facilities (5.1%) or going on a safari (27.3%). Direct interactions consisted of feeding, holding and/or touching non-domesticated animals (6.2%) and swimming with (5.2%) or riding (9.1%) non-domesticated animals. Respondents who visited captive facilities attended shows with non-domesticated animals (2.2%) and visited rehabilitation centres (1.1%) or species-specific farms and parks where non-domesticated animals could be viewed in captivity (1.9%). Those who went on a safari went diving or snorkelling (2.0%) and attended species specific excursions (5.9%) or general safaris to watch non-domesticated animals in the wild (19.4%).

### Photograph with wild cat species

#### Intention

**Fig 3:**
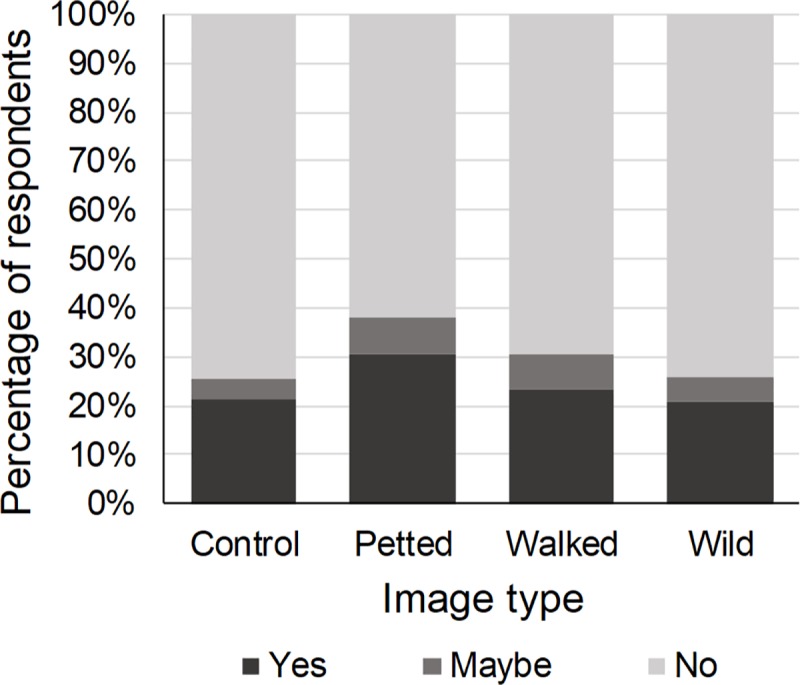
Percentage of respondents who would like to be photographed with wild cat species in relation to the type of image shown. Compared to the control image, respondents who were shown an image of a wild cat species being petted by a human were significantly more likely to have a desire to be photographed with wild cats.

Twenty-four percent of the respondents expressed a desire to be photographed with a wild cat species. The type of image shown to the respondents affected this desire (χ^2^ = 8.54, p = 0.04). Compared to the control image, respondents who were presented with an image in which a wild cat species was petted by a human were 1.77 times more likely to want to be photographed with wild cats (Table 1, Fig 3). The intention to be photographed with wild cats was not affected by the species in the image (χ^2^ = 3.73, p = 0.16) (Table 1). Previous engagement in WTAs influenced the desire to be photographed with wild cat species (χ^2^ = 7.62, p = 0.05). Compared to those who had not previously engaged in WTAs, respondents who had visited captive facilities were 2.53 times more likely to want to be photographed with wild cat species (Table 1). Gender (χ^2^ = 14.15, p < 0.01), age (χ^2^ = 31.05, p < 0.01) and education (χ^2^ = 8.56, p < 0.01) also affected this desire. Males, young adults (≤ 24) and lower educated respondents were more likely to want to be photographed with wild cats (Table 1).

**Table 1:** Test statistics and effect sizes for variables in the model to assess the respondents’ intention to be photographed with wild cat species. Parameter estimate (β), standard error (SE), Wald statistics (Wald), degrees of freedom (df), level of significance (p), cumulative odds ratio (Exp (β)) and 95% confidence interval of the cumulative odds ratio (95% CI Exp (β)) for variables in the model. The answer options no, maybe, yes were coded as 1, 2, 3.

*Intention to be photographed with a wild cat species*									
Variable	Category	β	SE	Wald	df	p	Exp (β)	95% CI Exp (β)	
								Lower	Upper
Gender (Female = reference)	Male	0.69	0.18	13.87	1	<0.01	1.99	1.38	2.85
Age (≥ 65 = reference)	≤ 24	1.33	0.34	14.92	1	<0.01	3.77	1.92	7.41
	25–44	0.32	0.36	0.77	1	0.38	1.38	0.68	2.81
	45–64	0.40	0.37	1.14	1	0.29	1.48	0.72	3.06
Education		-0.14	0.05	8.56	1	<0.01	0.87	0.79	0.95
Previous engagement in WTAs (None = reference)	Interaction	0.40	0.25	2.58	1	0.11	1.49	0.92	2.41
	Captivity	0.93	0.44	4.47	1	0.03	2.53	1.07	6.00
	Safari	-0.17	0.26	0.45	1	0.50	0.84	0.51	1.39
Cat species (Lion = reference)	Caracal	-0.30	0.22	1.75	1	0.19	0.74	0.48	1.15
	Cheetah	0.13	0.22	0.34	1	0.56	1.14	0.74	1.75
Image type (Control = reference)	Wild	-0.01	0.26	0.00	1	0.96	0.99	0.59	1.65
	Petted	0.57	0.26	4.98	1	0.03	1.77	1.07	2.93
	Walked	0.19	0.26	0.55	1	0.46	1.21	0.73	2.00

Significant results are highlighted in grey

#### Perception

**Fig 4:**
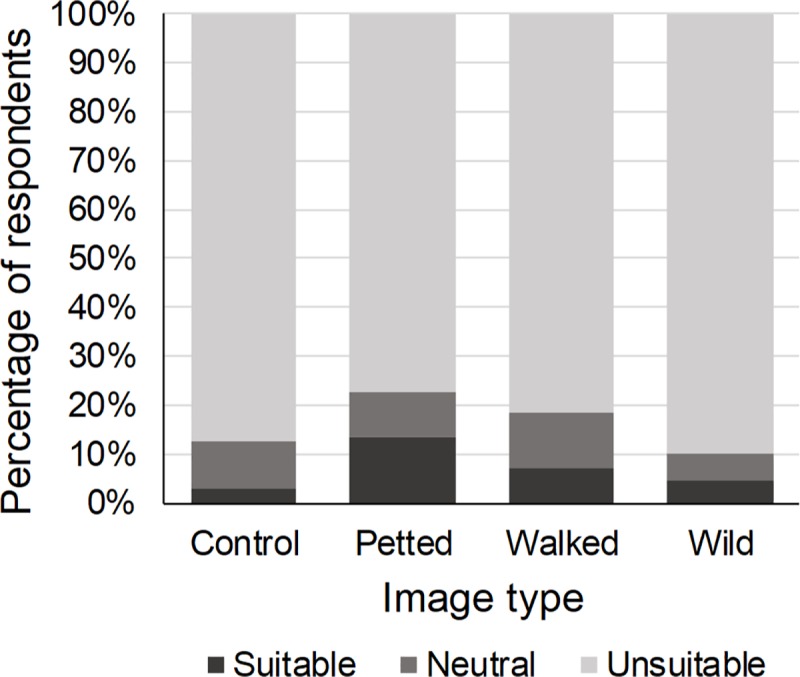
Percentage of respondents who perceived wild cat species as suitable to be photographed with tourists in relation to the type of image shown. Compared to the control image, respondents who were shown an image of a wild cat species being petted by a human were significantly more likely to perceive wild cats as suitable to be photographed with tourists.

Seven percent of the respondents perceived wild cats as suitable to be photographed with tourists. The type of image shown affected this perception (χ^2^ = 12.75, p < 0.01). Respondents who were presented with an image in which a wild cat species was petted by a human were 2.36 times more likely to perceive wild cats as suitable to be photographed with tourists (Table 2, Fig 4). The wild cat species in the image also affected the respondents’ perception of the suitability of wild cat species to be photographed with tourists (χ^2^ = 5.62, p = 0.05): respondents presented with an image of a caracal were 1.82 times more likely to perceive wild cats as suitable than those presented with an image of a cheetah or a lion (Table 2). Previous engagement in WTAs did not influence the respondents’ perception of the suitability of wild cat species to be photographed with tourists (χ^2^ = 4.11, p = 0.25). Gender (χ^2^ = 6.70, p = 0.01), age (χ^2^ = 14.70, p < 0.01) and education (χ^2^ = 10.61, p < 0.01) did: males, young adults (≤ 24) and lower educated respondents more often perceived wild cats as suitable for this tourist activity (Table 2).

**Table 2:** Test statistics and effect sizes for variables in the model to assess the respondents’ perception of the suitability of wild cat species to be photographed with tourists. Parameter estimate (β), standard error (SE), Wald statistics (Wald), degrees of freedom (df), level of significance (p), cumulative odds ratio (Exp (β)) and 95% confidence interval of the cumulative odds ratio (95% CI Exp (β)) for variables in the model. The answer options unsuitable, not suitable but also not unsuitable, suitable were coded as 1, 2, 3.

*Suitability wild cat species to be photographed with tourists*									
Variable	Category	B	SE	Wald	df	p	Exp (β)	95% CI Exp (β)	
								Lower	Upper
Gender (Female = reference)	Male	0.60	0.23	6.57	1	0.01	1.82	1.15	2.87
Age (≥ 65 = reference)	≤ 24	0.87	0.42	4.30	1	0.04	2.39	1.05	5.45
	25–44	-0.05	0.46	0.01	1	0.91	0.95	0.39	2.34
	45–64	-0.06	0.47	0.02	1	0.89	0.94	0.37	2.37
Education		-0.20	0.06	10.59	1	<0.01	0.82	0.72	0.92
Previous engagement in WTAs (None = reference)	Interaction	-0.59	0.35	2.83	1	0.09	0.55	0.28	1.10
	Captivity	-0.81	0.78	1.07	1	0.30	0.45	0.10	2.06
	Safari	-0.25	0.32	0.60	1	0.44	0.78	0.42	1.45
Cat species (Lion = reference)	Caracal	0.60	0.27	4.82	1	0.03	1.82	1.07	3.10
	Cheetah	0.11	0.30	0.13	1	0.72	1.11	0.62	2.01
Image type (Control = reference)	Wild	-0.20	0.36	0.32	1	0.57	0.82	0.40	1.65
	Petted	0.86	0.32	7.24	1	<0.01	2.36	1.26	4.40
	Walked	0.43	0.32	1.77	1	0.18	1.54	0.81	2.90

Significant results are highlighted in grey

Of the respondents who had the desire to be photographed with wild cat species, 18.1% felt wild cats are suitable to be photographed with tourists, while 68.4% believed wild cats are unsuitable for this tourist activity. Only a small percentage (2.6%) of the respondents who did not want to be photographed with wild cat species thought wild cats are suitable to be photographed with tourist, the majority (90.7%) believed wild cats are unsuitable for such a tourist activity.

### Walk with wild cat species

#### Intention

Eighteen percent of the respondents expressed a desire to walk with a wild cat species. The type of image (χ^2^ = 1.81, p = 0.61) or the cat species in the image (χ^2^ = 1.74, p = 0.42) did not affect this desire (Table 3). Previous engagement in WTAs did (χ^2^ = 9.65, p = 0.02): respondents who had visited captive facilities were 2.94 times more likely to have the desire to walk with a wild cat species than those who had not previously participated in WTAs (Table 3). Gender (χ^2^ = 17.52, p < 0.01), age (χ^2^ = 29.57, p < 0.01) and education (χ^2^ = 10.81, p < 0.01) also had a significant effect. Males, young adults (≤ 24) and lower educated respondents were more likely to want to walk with wild cats (Table 3).

**Table 3:** Test statistics and effect sizes for variables in the model to assess the respondents’ intention to walk with wild cat species. Parameter estimate (β), standard error (SE), Wald statistics (Wald), degrees of freedom (df), level of significance (p), cumulative odds ratio (Exp (β)) and 95% confidence interval of the cumulative odds ratio (95% CI Exp (β)) for variables in the model. The answer options no, maybe, yes were coded as 1, 2, 3.

*Intention to walk with a wild cat species*									
Variable		β	SE	Wald	df	p	Exp (β)	95% CI Exp (β)	
								Lower	Upper
Gender (Female = reference)	Male	0.85	0.21	16.94	1	<0.01	2.33	1.56	3.48
Age (≥ 65 = reference)	≤ 24	1.66	0.43	14.69	1	<0.01	5.24	2.25	12.21
	25–44	0.79	0.45	3.00	1	0.08	2.19	0.90	5.33
	45–64	0.63	0.46	1.82	1	0.18	1.87	0.75	4.64
Education		-0.18	0.05	10.79	1	<0.01	0.84	0.75	0.93
Previous engagement in WTAs (None = reference)	Interaction	0.42	0.26	2.53	1	0.11	1.52	0.91	2.53
	Captivity	1.08	0.44	6.02	1	0.01	2.94	1.24	6.94
	Safari	-0.28	0.30	0.88	1	0.35	0.76	0.43	1.35
Cat species (Lion = reference)	Caracal	-0.01	0.24	0.00	1	0.98	0.99	0.63	1.57
	Cheetah	-0.30	0.26	1.36	1	0.24	0.74	0.45	1.22
Image type (Control = reference)	Wild	-0.01	0.29	0.00	1	0.97	0.99	0.56	1.73
	Petted	0.32	0.28	1.24	1	0.27	1.37	0.79	2.40
	Walked	0.16	0.28	0.31	1	0.58	1.17	0.67	2.04

Significant results are highlighted in grey

#### Perception

**Fig 5:**
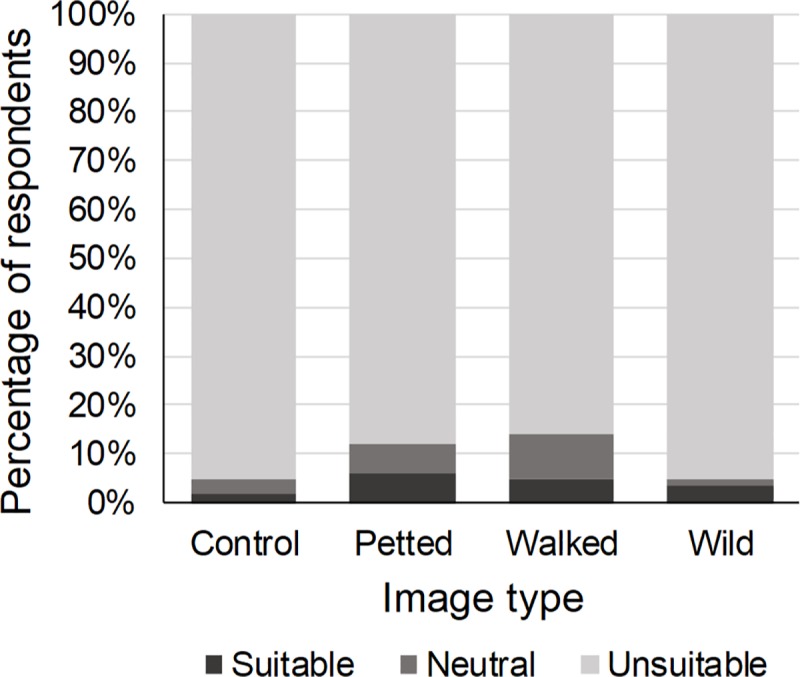
Percentage of respondents who perceived wild cat species as suitable to walk with tourists in relation to the type of image shown. Compared to the control image, respondents who were shown an image of a wild cat species being petted or walked by a human were significantly more likely to perceive wild cat species as suitable to walk with tourists.

Five percent of the respondents perceived wild cat species as suitable to walk with tourists. The type of image shown affected this perception (χ^2^ = 12.59, p < 0.01). Compared to those presented with a control image, respondents who were presented with an image of a wild cat being petted by a human were 2.67 times more likely to perceive wild cats as suitable to walk with tourists, while those presented with an image of a wild cat being walked by a human were 2.98 times more likely to perceive wild cats as suitable for this tourist activity (Table 4, Fig 5). The wild cat species in the image also affected the respondents’ perception of the suitability of wild cat species to walk with tourists (χ^2^ = 15.13, p < 0.01). Respondents who were presented with an image of a caracal were 2.84 times more likely to perceive wild cats as suitable for this tourist activity than those presented with an image of a cheetah or a lion (Table 4). Previous engagement in WTAs did not influence the respondents’ perception of the suitability of wild cats to walk with tourists, neither did gender, age or education (1.21 ≤ χ^2^ ≤ 5.33, 0.07 ≤ p ≤ 0.38) (Table 4).

**Table 4:** Test statistics and effect sizes for variables in the model to assess the respondents’ perception of the suitability of wild cat species to walk with tourists. Parameter estimate (β), standard error (SE), Wald statistics (Wald), degrees of freedom (df), level of significance (p), cumulative odds ratio (Exp (β)) and 95% confidence interval of the cumulative odds ratio (95% CI Exp (β)) for variables in the model. The answer options unsuitable, not suitable but also not unsuitable, suitable were coded as 1, 2, 3.

*Suitability wild cat species to walk with tourists*									
Variable		β	SE	Wald	df	p	Exp (β)	95% CI Exp (β)	
								Lower	Upper
Gender (Female = reference)	Male	0.54	0.30	3.21	1	0.07	1.71	0.95	3.08
Age (≥ 65 = reference)	≤ 24	0.79	0.57	1.88	1	0.17	2.19	0.71	6.73
	25–44	0.22	0.61	0.14	1	0.71	1.25	0.38	4.13
	45–64	0.00	0.64	0.00	1	0.99	1.00	0.29	3.48
Education		-0.09	0.08	1.22	1	0.27	0.92	0.78	1.07
Previous engagement in WTAs (None = reference)	Interaction	-0.12	0.39	0.10	1	0.75	0.88	0.41	1.91
	Captivity	-1.00	1.06	0.90	1	0.34	0.37	0.05	2.91
	Safari	-0.62	0.43	2.05	1	0.15	0.54	0.23	1.25
Cat species (Lion = reference)	Caracal	1.04	0.35	8.71	1	<0.01	2.84	1.42	5.67
	Cheetah	-0.19	0.44	0.19	1	0.66	0.82	0.35	1.96
Image type (Control = reference)	Wild	-0.05	0.52	0.01	1	0.92	0.95	0.34	2.66
	Petted	0.98	0.45	4.72	1	0.03	2.67	1.10	6.48
	Walked	1.09	0.44	6.18	1	0.01	2.98	1.26	7.06

Significant results are highlighted in grey

Of the respondents who had a desire to walk with wild cat species, 12.7% believed wild cats are suitable to walk with tourists, while 76.3% believed wild cats are unsuitable for this tourist activity. A very small percentage (2.0%) of the respondents who did not want to walk with wild cat species felt wild cats are suitable to walk with tourists, the majority (96.0%) believed wild cats are unsuitable for such a tourist activity.

### Pet wild cat species

#### Intention

**Fig 6:**
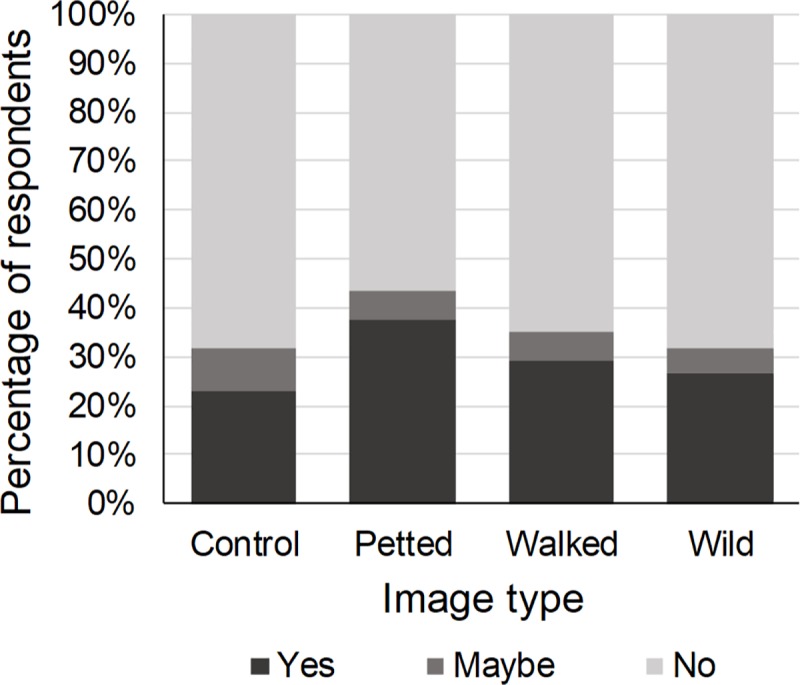
Percentage of respondents who would like to pet a wild cat species in relation to the type of image shown. Compared to the control image, respondents who were shown an image of a wild cat species being petted by a human were significantly more likely to have a desire to pet wild cat species.

Twenty-nine percent of the respondents expressed a desire to pet a wild cat species. This desire was affected by the type of image shown (χ^2^ = 8.80, p = 0.03): respondents who were presented with an image of a wild cat species being petted by a human were 1.91 times more likely to want to pet wild cats (Table 5, Fig 6). The intention to pet wild cats was not affected by the species in the image (χ^2^ = 0.29, p = 0.87) (Table 5). Previous engagement in WTAs did influence the respondents’ desire to pet wild cats (χ^2^ = 8.27, p = 0.04). Respondents who had visited captive facilities were 2.41 times more likely to want to pet wild cat species than those who had not previously participated in WTAs (Table 5). Gender (χ^2^ = 16.51, p < 0.01), age (χ^2^ = 48.88, p < 0.01) and education (χ^2^ = 9.50, p < 0.01) also influenced the intention to pet wild cats. Males, young adults (≤ 24) and lower educated respondents were more likely to want to pet wild cats (Table 5).

**Table 5:** Test statistics and effect sizes for variables in the model to assess the respondents’ intention to pet a wild cat species. Parameter estimate (β), standard error (SE), Wald statistics (Wald), degrees of freedom (df), level of significance (p), cumulative odds ratio (Exp (β)) and 95% confidence interval of the cumulative odds ratio (95% CI Exp (β)) for variables in the model. The answer options no, maybe, yes were coded as 1, 2, 3.

*Intention to pet a wild cat species*									
Variable		β	SE	Wald	df	p	Exp (β)	95% CI Exp (β)	
								Lower	Upper
Gender (Female = reference)	Male	0.72	0.18	16.15	1	<0.01	2.06	1.45	2.92
Age (≥ 65 = reference)	≤ 24	1.34	0.32	17.89	1	<0.01	3.82	2.05	7.10
	25–44	0.43	0.33	1.74	1	0.19	1.54	0.81	2.93
	45–64	-0.18	0.35	0.26	1	0.61	0.84	0.42	1.67
Education		-0.15	0.05	9.47	1	<0.01	0.86	0.79	0.95
Previous engagement in WTAs (None = reference)	Interaction	0.33	0.24	1.89	1	0.17	1.39	0.87	2.23
	Captivity	0.88	0.44	4.01	1	0.04	2.41	1.02	5.69
	Safari	-0.30	0.25	1.47	1	0.23	0.74	0.45	1.20
Cat species (Lion = reference)	Caracal	-0.11	0.21	0.28	1	0.60	0.89	0.59	1.35
	Cheetah	-0.07	0.22	0.10	1	0.76	0.94	0.61	1.43
Image type (Control = reference)	Wild	0.02	0.25	0.01	1	0.94	1.02	0.62	1.67
	Petted	0.65	0.25	6.81	1	<0.01	1.91	1.17	3.10
	Walked	0.18	0.25	0.53	1	0.47	1.20	0.74	1.95

Significant results are highlighted in grey

#### Perception

**Fig 7:**
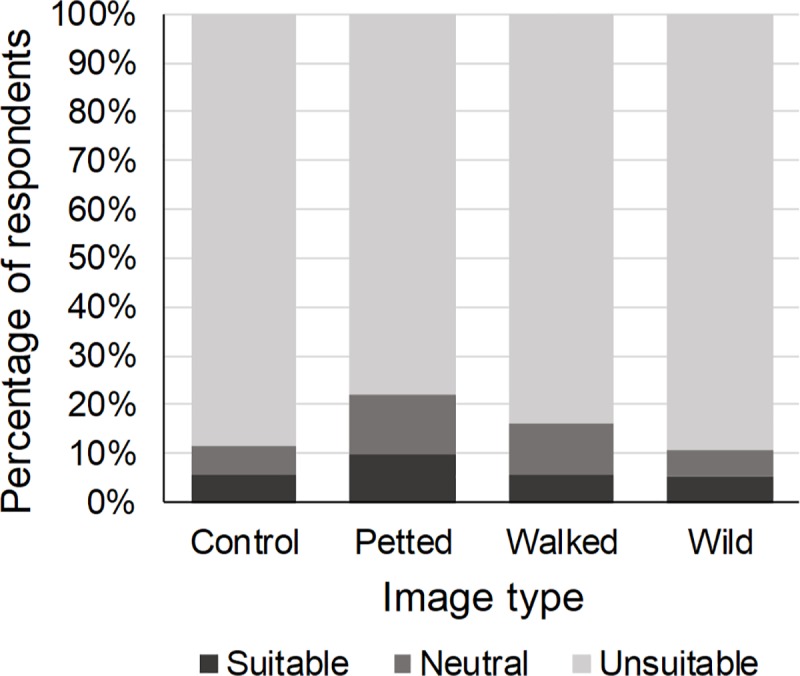
Percentage of respondents who perceived wild cat species as suitable to be petted by tourists in relation to the type of image shown. Compared to the control image, respondents who were shown an image of a wild cat species being petted by a human were significantly more likely to perceive wild cat species as suitable to be petted by tourists.

Seven percent of the respondents perceived wild cat species as suitable to be petted by tourists. The respondents’ perception of the suitability of wild cat species for this tourist activity was affected by the type of image shown (χ^2^ = 9.69, p = 0.02). Respondents who were presented with an image of a wild cat species being petted by a human were 2.19 times more likely to perceive wild cats as suitable to be petted by tourists (Table 6, Fig 7). The wild cat species in the image did not influence the likelihood respondents perceived wild cats as suitable to be petted by tourists (χ^2^ = 5.71, p = 0.06), neither did previous engagement in WTAs (χ^2^ = 5.18, p = 0.16) (Table 6). Gender (χ^2^ = 7.60, p < 0.01), age (χ^2^ = 18.99, p < 0.01) and education (χ^2^ = 6.02, p = 0.01) did: males, young adults (≤ 24) and lower educated respondents more often perceived wild cats as suitable to be petted by tourists (Table 6).

**Table 6:** Test statistics and effect sizes for variables in the model to assess the respondents’ perception of the suitability of wild cat species to be petted by tourists. Parameter estimate (β), standard error (SE), Wald statistics (Wald), degrees of freedom (df), level of significance (p), cumulative odds ratio (Exp (β)) and 95% confidence interval of the cumulative odds ratio (95% CI Exp (β)) for variables in the model. The answer options unsuitable, not suitable but also not unsuitable, suitable were coded as 1, 2, 3.

*Suitability wild cat species to be petted by tourists*									
Variable		β	SE	Wald	df	p	Exp (β)	95% CI Exp (β)	
								Lower	Upper
Gender (Female = reference)	Male	0.65	0.24	7.40	1	<0.01	1.92	1.20	3.07
Age (≥ 65 = reference)	≤ 24	0.98	0.44	5.01	1	0.03	2.68	1.13	6.34
	25–44	0.00	0.48	0.00	1	0.99	1.00	0.39	2.56
	45–64	-0.19	0.51	0.14	1	0.71	0.83	0.31	2.24
Education		-0.16	0.06	6.04	1	0.01	0.86	0.76	0.97
Previous engagement in WTAs (None = reference)	Interaction	0.20	0.31	0.43	1	0.51	1.22	0.67	2.24
	Captivity	-1.34	1.05	1.62	1	0.20	0.26	0.03	2.06
	Safari	-0.47	0.35	1.87	1	0.17	0.62	0.32	1.23
Cat species (Lion = reference)	Caracal	0.31	0.27	1.34	1	0.25	1.37	0.81	2.32
	Cheetah	-0.39	0.31	1.55	1	0.21	0.68	0.37	1.25
Image type (Control = reference)	Wild	-0.14	0.36	0.15	1	0.70	0.87	0.43	1.77
	Petted	0.79	0.33	5.75	1	0.02	2.19	1.15	4.16
	Walked	0.34	0.34	1.00	1	0.32	1.40	0.72	2.72

Significant results are highlighted in grey

Of the respondents who had a desire to pet wild cat species, 17.2% felt wild cats are suitable to be petted by tourists while 61.8% believed wild cats are unsuitable for such tourist activity. Very few (1.2%) respondents who were not inclined to pet wild cat species thought wild cats are suitable to be petted by tourists, the majority (96.4%) believed wild cats are unsuitable for such a tourist activity.

### Keep wild cat species as a pet

Seven percent of the respondents believed a wild cat species might be a nice pet. Whether or not respondents perceived wild cats as potentially nice pets was not affected by image type (χ^2^ = 4.55, p = 0.21) nor the wild cat species in the image (χ^2^ = 0.21, p = 0.90) (Table 7). Previous engagement in WTAs also did not influence whether respondents perceived wild cats as potentially nice pets, neither did gender or age of the respondents (0.47 ≤ χ^2^ ≤ 2.87, 0.32 ≤ p ≤ 0.47) (Table 7). Education did affect whether or not respondents thought wild cat species might be nice pets (χ^2^ = 8.42, p < 0.01): lower educated respondents were more likely to perceive wild cats as potentially nice pets (Table 7).

**Table 7:** Test statistics and effect sizes for variables in the model to assess whether respondents perceived a wild cat species as a potentially nice pet. Parameter estimate (β), standard error (SE), Wald statistics (Wald), degrees of freedom (df), level of significance (p), odds ratio (Exp (β)) and 95% confidence interval of the odds ratio (95% CI Exp (β)) for variables in the model. The answer option no (not a nice pet) was used as the reference category.

*Perception of a wild cat species as a nice pet (Not a nice pet = reference)*									
Variable		β	SE	Wald	df	p	Exp (β)	95% CI Exp (β)	
								Lower	Upper
Gender (Female = reference)	Male	0.33	0.33	1.00	1	0.32	1.39	0.73	2.67
Age (≥ 65 = reference)	≤ 24	0.96	0.77	1.57	1	0.21	2.61	0.58	11.74
	25–44	0.71	0.81	0.77	1	0.38	2.03	0.42	9.87
	45–64	1.06	0.79	1.79	1	0.18	2.88	0.61	13.59
Education		-0.25	0.09	8.32	1	<0.01	0.78	0.66	0.92
Previous engagement in WTAs (None = reference)	Interaction	0.57	0.40	2.07	1	0.15	1.77	0.81	3.84
	Captivity	-0.38	1.06	0.13	1	0.72	0.68	0.09	5.46
	Safari	-0.24	0.52	0.21	1	0.65	0.79	0.28	2.19
Cat species (Lion = reference)	Caracal	0.15	0.39	0.15	1	0.70	1.17	0.54	2.51
	Cheetah	-0.00	0.42	0.00	1	0.99	1.00	0.44	2.26
Image type (Control = reference)	Wild	0.88	0.55	2.55	1	0.11	2.42	0.82	7.15
	Petted	1.03	0.55	3.55	1	0.06	2.81	0.96	8.20
	Walked	0.88	0.55	2.54	1	0.11	2.41	0.82	7.08

Significant results are highlighted in grey

Very few respondents actually believed wild cat species are suitable to be kept as pets (1.4%), however, respondents who thought wild cats might be nice pets more often believed wild cats are suitable to be kept as pets than those who thought wild cats are not nice pets (14.3% versus 0.5%).

### Danger wild cat species represent

**Fig 8:**
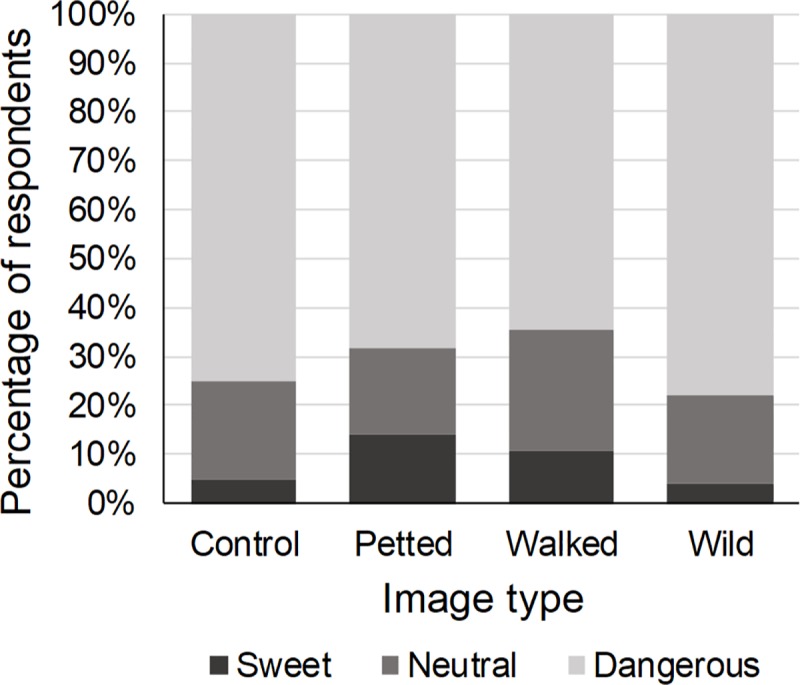
Percentage of respondents who perceived wild cat species as sweet, not sweet but also not dangerous (neutral) or dangerous in relation to the type of image shown. Compared to the control image, respondents who were shown an image of a wild cat species being petted by a human were significantly less likely to perceive wild cat species as dangerous.

Eight percent of the respondents perceived wild cat species as sweet, 20.1% as not sweet but also not dangerous (neutral). Image type affected the respondents’ perception of the danger wild cat species represent (χ^2^ = 18.61, p < 0.01). Respondents who were presented with an image of a wild cat being petted by a human were 3.23 times more likely to perceive wild cats as sweet (Table 8, Fig 8). The perceived danger was also affected by the species in the image (χ^2^ = 12.06, p = 0.02): compared to cheetah and lion, respondents were 1.78 times more likely to perceive caracal as neutral. Previous engagement in WTAs did not influence perception of the danger wild cat species represent (χ^2^ = 6.30, p = 0.39) (Table 8). The respondents’ gender did (χ^2^ = 20.52, p < 0.01): males were more likely to perceive wild cat species as neutral (Table 8). The respondents’ age (χ^2^ = 9.43, p = 0.15) and education (χ^2^ = 0.36, p = 0.84) did not affect the perceived danger wild cat species represent (Table 8).

**Table 8:** Test statistics and effect sizes for variables in the model to assess the respondents’ perception of the danger wild cat species represent. Parameter estimate (β), standard error (SE), Wald statistics (Wald), degrees of freedom (df), level of significance (p), odds ratio (Exp (β)) and 95% confidence interval of the odds ratio (95% CI Exp (β)) for variables in the model. The answer option dangerous was used as the reference category.

*Danger wild cat species represent (Dangerous = reference)*									
Variable		β	SE	Wald	df	p	Exp (β)	95% CI Exp (β)	
								Lower	Upper
*Neutral (not sweet but also not dangerous)*									
Gender (Female = reference)	Male	0.94	0.22	18.58	1	<0.01	2.55	1.67	3.90
Age (≥ 65 = reference)	≤ 24	0.60	0.36	2.72	1	0.10	1.82	0.89	3.69
	25–44	0.10	0.38	0.07	1	0.80	1.10	0.53	2.30
	45–64	-0.18	0.40	0.21	1	0.64	0.83	0.38	1.82
Education		-0.00	0.06	0.00	1	0.98	1.00	0.89	1.12
Previous engagement in WTAs (None = reference)	Interaction	0.33	0.28	1.46	1	0.23	1.40	0.81	2.40
	Captivity	-1.08	0.76	1.99	1	0.16	0.34	0.08	1.52
	Safari	-0.24	0.29	0.70	1	0.40	0.79	0.45	1.38
Cat species (Lion = reference)	Caracal	0.58	0.25	5.50	1	0.02	1.78	1.10	2.88
	Cheetah	-0.24	0.27	0.79	1	0.37	0.78	0.46	1.34
Image type (Control = reference)	Wild	-0.21	0.30	0.53	1	0.47	0.81	0.45	1.44
	Petted	-0.09	0.31	0.09	1	0.76	0.91	0.50	1.66
	Walked	0.37	0.28	1.72	1	0.19	1.45	0.83	2.53
*Sweet*									
Gender (Female = reference)	Male	-0.08	0.31	0.07	1	0.80	0.92	0.51	1.68
Age (≥ 65 = reference)	≤ 24	0.48	0.54	0.79	1	0.37	1.62	0.56	4.64
	25–44	0.03	0.57	0.00	1	0.96	1.03	0.34	3.13
	45–64	0.16	0.57	0.08	1	0.77	1.18	0.39	3.59
Education		-0.05	0.08	0.36	1	0.55	0.95	0.82	1.12
Previous engagement in WTAs (None = reference)	Interaction	0.00	0.42	0.00	1	0.99	1.00	0.44	2.26
	Captivity	-0.34	0.79	0.19	1	0.67	0.71	0.15	3.35
	Safari	-0.41	0.43	0.92	1	0.34	0.66	0.29	1.54
Cat species (Lion = reference)	Caracal	0.39	0.36	1.20	1	0.27	1.48	0.73	2.97
	Cheetah	-0.16	0.39	0.16	1	0.69	0.86	0.40	1.83
Image type (Control = reference)	Wild	-0.23	0.54	0.19	1	0.67	0.79	0.28	2.27
	Petted	1.17	0.44	7.06	1	<0.01	3.23	1.36	7.67
	Walked	0.86	0.46	3.51	1	0.06	2.35	0.96	5.76

Significant results are highlighted in grey

**Fig 9:**
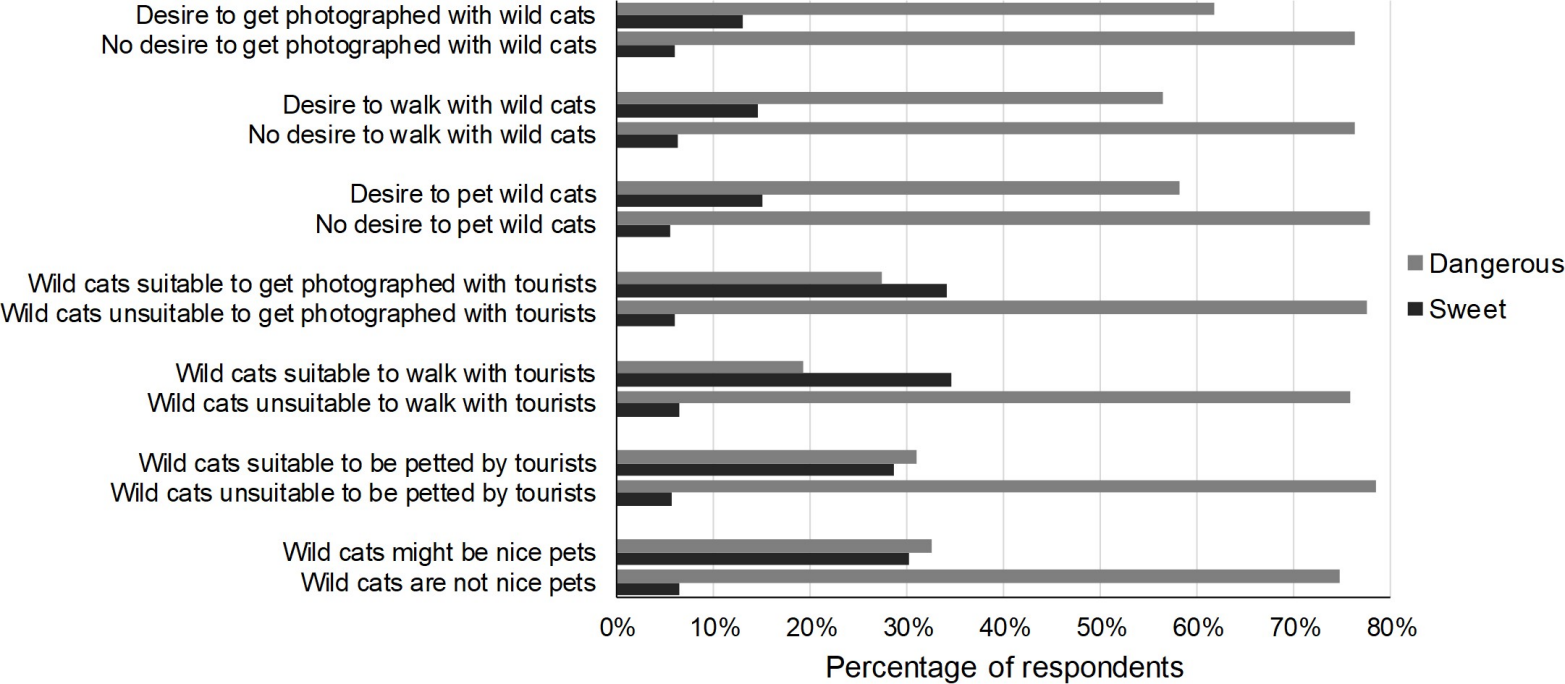
Percentage of respondents who perceived wild cat species as sweet or dangerous in relation to their intention to be photographed with, walk with or pet wild cats and their perception of the suitability of wild cats for such tourist activities. Respondents who expressed a desire to be photographed with, walk with or pet wild cats and/or respondents who thought wild cats are suitable for such activities with tourists more often perceived wild cats as sweet than those who did not.

Compared to respondents who believed wild cat species are dangerous, respondents who perceived wild cat species as sweet were more likely to express a desire to be photographed with (χ^2^ = 10.15, p < 0.01), walk with (χ^2^ = 12.85, p < 0.01) or pet wild cats (χ^2^ = 20.05, p < 0.01) and more often felt wild cat species are suitable for such tourist activities (suitable to be photographed with tourists: χ^2^ = 67.30, p < 0.01, walk with tourists: χ^2^ = 50.21, p < 0.01, be petted by tourists: χ^2^ = 49.69, p < 0.01). Respondents who thought wild cat species are sweet were also more likely to perceive wild cats as possible nice pets (χ^2^ = 45.98, p < 0.01) (Fig 9).

### Conservation status

Eleven percent of the respondents believed wild cat species are not endangered in the wild, the majority of the respondents thought wild cats are either endangered (71.2%) or very endangered (17.7%). The type of image shown (χ^2^ = 3.11, p = 0.38) or the wild cat species in the image (χ^2^ = 4.89, p = 0.09) did not affect the respondents’ perception of the conservation status of wild cats (Table 9). Neither did previous engagement in WTAs, gender, age or level of education (0.01 ≤ χ^2^ ≤ 4.43, 0.22 ≤ p ≤ 0.93) (Table 9).

**Table 9:** Test statistics and effect sizes for variables in the model to assess the respondents’ perception of the conservation status of wild cat species. Parameter estimate (β), standard error (SE), Wald statistics (Wald), degrees of freedom (df), level of significance (p), cumulative odds ratio (Exp (β)) and 95% confidence interval of the cumulative odds ratio (95% CI Exp (β)) for variables in the model. The answer options not endangered, endangered, very endangered were coded as 1, 2, 3.

*Perception of the conservation status of wild cat species*									
Variable		β	SE	Wald	df	p	Exp (β)	95% CI Exp (β)	
								Lower	Upper
Gender (Female = reference)	Male	-0.19	0.18	1.16	1	0.28	0.83	0.58	1.17
Age (≥ 65 = reference)	≤ 24	0.08	0.30	0.06	1	0.81	1.08	0.59	1.96
	25–44	0.14	0.31	0.21	1	0.65	1.15	0.63	2.12
	45–64	0.42	0.32	1.77	1	0.18	1.53	0.82	2.85
Education		-0.00	0.05	0.01	1	0.93	1.00	0.91	1.10
Previous engagement in WTAs (None = reference)	Interaction	-0.36	0.25	2.03	1	0.16	0.70	0.43	1.14
	Captivity	-0.67	0.46	2.12	1	0.15	0.51	0.21	1.26
	Safari	0.09	0.24	0.14	1	0.71	1.09	0.69	1.74
Cat species (Lion = reference)	Caracal	-0.31	0.22	2.00	1	0.16	0.73	0.48	1.13
	Cheetah	0.17	0.22	0.64	1	0.42	1.19	0.78	1.81
Image type (Control = reference)	Wild	-0.26	0.24	1.17	1	0.28	0.77	0.48	1.24
	Petted	-0.31	0.26	1.44	1	0.23	0.74	0.45	1.21
	Walked	-0.42	0.25	2.89	1	0.09	0.66	0.40	1.07

## Discussion

We are repeatedly exposed to images of non-domesticated animals in books, movies and (social) media; the way we perceive the animals in those images is affected by how they are portrayed [35–39,71]. In (social) media, non-domesticated animals are regularly portrayed in interactions with humans (e.g. [42–49]). Although not empirically tested, it has been suggested that exposure to such images results in people becoming too familiar with non-domesticated animals, up to the point where they lose their instinctive fear and engage in dangerous behaviour [50,72] like taking selfies with wild animals [50,73,74], getting out of their car in a drive-through cheetah enclosure [72] or trying to stroke a wild lion on a safari [75]. In addition, portraying non-domesticated animals in interactions with humans may encourage participation in WTAs which provide us with the opportunity to engage in such interactions ourselves, at the expense of animal welfare, human safety and conservation [7–9,22,23,58,59,76].

This study shows portrayal of wild cat species interacting with humans affects people’s desire to engage in such interactions themselves and distorts their perception of the suitability of wild cats to be used for WTAs in which tourists interact with wild cats. Presenting people with an image of a wild cat species being petted by a human increased people’s desire to be photographed with or pet wild cats and made people more than twice (2.19–2.67) as likely to perceive wild cats as suitable for WTAs in which tourists are being photographed with, walk with or pet wild cats. The image of a wild cat species being walked by a human did not affect people’s desire to interact with wild cats, however, this image did make people almost three times (2.89) more likely to perceive wild cats as suitable to walk with tourists. Portrayal of a wild cat species interacting with humans did also influence people’s perception of the danger wild cats represent. The image of a wild cat species being petted by a human made people more than three times (3.23) as likely to perceive wild cats as sweet. People who perceived wild cats as sweet were more likely to want to interact with wild cats, consider wild cats suitable for such interactions and perceive wild cats as possible pets. However, contrary to a comparable study by Ross et al [37] on portrayal of chimpanzees, portrayal of wild cat species with humans did not directly affect attractiveness as a pet or perceived conservation status in the wild. This could be the result of species-specific perceptions but may also result from a higher level of awareness of our respondents as, compared to Ross et al’s study, a very high percentage of respondents perceived wild cats as unappealing/unsuitable pets and (very) endangered in the wild. Portraying wild cat species in a wild natural environment elicited the most neutral response: compared to the control image, the image of a wild cat species in the wild did not significantly influence people’s desire to interact with wild cats nor people’s perception of its suitability to interact with tourists, the danger it represents, suitability as a pet or its conservation status in the wild.

Mental representation of behaviour promotes engagement in this behaviour, therefore, humans tend to act in the same way they see others act [51]. This mechanism is likely to play a role when exposure to (social) media encourages engagement in certain behaviours [28,29] but may also explain the increased desire of respondents to interact with wild cats after visiting captive facilities to view non-domesticated animals. Respondents who visited such facilities attended shows with wild animals and visited rehabilitation centres or species-specific farms and parks. At these shows and venues, it is not unusual to see staff members interact with non-domesticated animals (e.g. [77,78]), therefore this may trigger a desire to engage in such interactions ourselves. Unlike those respondents who had participated in direct interactions with wild animals, this desire remained unsatisfied and respondents who visited captive facilities may therefore have been more inclined to engage in interactions with non-domesticated animals. Previous engagement in WTAs in which respondents interacted with non-domesticated animals did not affect the desire to interact with wild cat species. People who participated in WTAs are generally not aware of the negative animal welfare and conservation consequences this may have [9]. In this study, previous engagement in WTAs did not affect perceived suitability of wild cats to interact with tourists. The fact that engagement in interactions with non-domesticated animals in the past did not increase the desire to engage in interactions with wild cats in future, is therefore unlikely to be the result of an increased awareness of the negative consequences of such WTAs.

The demographic variables gender, age and education affected the desire to interact with wild cat species, perceived suitability of wild cats for WTAs, the danger wild cat species represent and the attractiveness of wild cats as pets. Age had the strongest impact: young adults were four to five times more likely to want to interact with wild cat species and two to three times more likely to perceive wild cats as suitable to be photographed with or petted by tourists. Compared to females, males were twice as likely to have the intention to engage in interactions with wild cat species and perceived wild cats as less dangerous and more suitable to interact with tourists. These findings are in line with other studies which have shown young adults and males are less fearful of carnivores [66,68] and more inclined to view animals as beneficial to human interest (practical and recreational value) [65,67]. Although the effect of gender and age was considerably stronger, level of education also played a role: lower educated people were more inclined to interact with wild cat species and more likely to perceive wild cats as suitable to interact with tourists. Lower educated people also more often perceived wild cat species as possible nice pets. Although fear of carnivores has been found to decrease with level of education [66,68], lower educated people have a less affective and emotional response to animals and, as supported by our findings, are more likely to view animals in light of their benefit to human interest [67].

Humans prefer certain animal species over others, e.g. big cats are popular with the public [71,79] and both lion and cheetah are among the ten most charismatic species in the world [80]. A species’ popularity is affected by several factors, for example size, attractiveness, danger to humans, rarity and the media attention it receives [11,71,79]. Although the wild cat species in the images used in this study varied considerably in size, danger to humans, and media attention, species did not affect people’s intention to engage in interactions with wild cats. However, species did affect people’s perception of wild cats: possibly due to their smaller size, caracal was perceived as less dangerous and more suitable to be photographed with or walked by tourists than cheetah or lion.

People who had a desire to engage in interactions with wild cat species mostly wanted to pet wild cats, followed by being photographed with or walk with wild cats. Despite the fact that forced proximity to humans can be stressful for non-domesticated animals [17–21], such wild cat-human interactions are commonly offered as WTAs [7,54–59]. The wild cat tourism industry is associated with removing cubs from their mothers at a very young age, wild cats being sold as hunting trophies to the canned hunting industry, high incidences of attacks on humans and no conservation return even though these wild cat WTAs are offered under the banner of conservation [7,22,23,58,59,76]. This is why animal welfare organisations try to raise awareness and discourage participation in these WTAs (e.g. [22,23]). In this study, most people were aware of the fact that wild cat species are unsuitable for WTAs, however, this did not necessarily stop them from having the desire to engage in such activities: 62–76% of the people who wanted to interact with wild cats realized wild cat species are unsuitable for such interactions. More than twenty percent of the people had in the past participated in WTAs which involved direct interactions with wildlife. Even though respondents may have been aware non-domesticated animals are unsuitable for WTAs, they may not have realized how serious the negative welfare and conservation implications can be [9]. The moral concerns about such activities may therefore not have been high enough to override the desire to interact with animals. As many as 91–96% of the respondents who had no desire to interact with wild cat species were aware wild cats are unsuitable to interact with humans. Although awareness campaigns may not be as effective as hoped, this does suggest raising awareness discourages people to interact with non-domesticated animals.

## Conclusion

Most people do not have the opportunity to watch non-domesticated animals in their natural environment and rely on (social) media to learn about these animals [26,27].

The way in which (social) media portrays non-domesticated animals affects our perception of and behaviour to these animals [26,35–40]. This places an important responsibility on (social) media producers to correctly represent animals and their relationship to humans. Today, (social) media increasingly portrays non-domesticated animals in interactions with humans [27,40,41]. Our study shows this can increase people’s desire to engage in interactions with non-domesticated animals themselves, reduce objections against the exploitation of non-domesticated animals for such interactions and distort people’s perception of the danger wild animals represent.

An increased desire to interact with non-domesticated animals does not necessarily mean people will immediately act on this desire, if only because practical and financial considerations prevent them from doing so. However, continuous exposure to images of human-wildlife interactions may result in people coming to perceive wild animals as entities available for human entertainment as opposed to interesting and priceless creatures in their own right [27,50,67]. This is likely to lower the threshold to engage in interactions with non-domesticated animals in future and reduce moral concerns about the exploitation of wild animals for such a purpose [67]. Although further research is necessary to determine what the long-term behavioural consequences of exposure to images of non-domesticated animals in interactions with humans are, studies have shown frequent exposure to media images can result in undesirable long-term behavioural change [28,31,81]. It is therefore important for (social) media producers and others who use images of wild animals to think carefully about the way in which they portray an animal and to be aware of the attitude, perceptions and behaviour this portrayal may promote. Failure to do so could unintentionally encourage unethical, irresponsible and even dangerous behaviour with often dire consequences for both animals and humans involved.

Male, lower educated, young adults are more likely to view animals in light of their benefit to human kind and therefore particularly inclined to engage in interactions with non-domesticated animals. Apart from zoo visits (60.0%), the majority of this demographic group (68.0%) received information about wild animals by watching wildlife series on animal dedicated cable television networks (36.0%) and, to a lesser extent, Dutch television channels (18.0%). Through careful consideration of how animals are portrayed in wildlife series popular with this demographic group, it may be possible to promote a more moralistic attitude [67] towards wildlife under male, lower educated, young adults. In addition, awareness campaigns by animal welfare organisations and others should target this demographic group in particular to emphasize the negative animal welfare and conservation consequences and potential danger of participation in WTAs which offer the opportunity to interact with wildlife. Although the desire to interact with non-domesticated animals can be stronger than the moral obligation not to, awareness campaigns do seem to discourage people from interacting with wild animals through detrimental WTAs [82].

## Supporting information

S1 Appendix Questionnaire in English and Dutch. English (translated from Dutch) and Dutch questionnaire used for the interviews to assess the respondent’s intention to be photographed with, pet or walk with wild cat species, their perception of the suitability of wild cat species to be used for such tourist activities, the danger wild cats represent, the suitability of wild cats as pets and their conservation status in the wild in relation to the type of image shown and the wild cat species in the image.
(DOCX)
