# Supplementary material for: I thought I saw a pussy cat: Portrayal of wild cats in friendly interactions with humans distorts perceptions and encourages interactions with wild cat species
Source: PLoS One. 2019 May 1;14(5):e0215211. doi: 10.1371/journal.pone.0215211 (PMC6493739; doi:10.1371/journal.pone.0215211)
Supplement: S1 Appendix — English (translated from Dutch) and Dutch questionnaire used for the interviews to assess the respondent’s intention to be photographed with, pet or walk with wild cat species, their perception of the suitability of wild cat species to be used for such tourist activities, the danger wild cats represent, the suitability of wild cats as pets and their conservation status in the wild in relation to the type of image shown and the wild cat species in the image. (DOCX) [file pone.0215211.s001.docx]

**S1 Appendix. Questionnaire in English and Dutch.** English (translated from Dutch) and Dutch questionnaire used for the interviews to assess the respondent’s intention to be photographed with, pet or walk with wild cat species, their perception of the suitability of wild cat species to be used for such tourist activities, the danger wild cats represent, the suitability of wild cats as pets and their conservation status in the wild in relation to the type of image shown and the wild cat species in the image.

| **Photograph used in the interview** | | | | | | | | | | | |
| --- | --- | --- | --- | --- | --- | --- | --- | --- | --- | --- | --- |
| **0 Caracal** | | **0 Cheetah** | | | | | **0 Lion** | | | |  |
| **0 Control** | | **0 Walking** | | | | | **0 Petting** | | | | **0 Wild** |
| Date: | | Location: | | | | | Name interviewer: | | | | |
| Gender respondent: | | | 0 Male | | 0 Female | | | |  |  | |
| What is your age? | | | | |  | | | |  |  | |
| What is the highest level of education you have completed? | | | | | | | | | 0 VMBO | 0 HAVO | |
| 0 VWO | 0 MBO | | 0 HBO | | 0 WO | | | | 0 Other: | | |
| In which village/town/city do you reside: | | | | | | | | |  |  | |
| 1a. Are you in a profession in which you have contact with animals: | | | | | | | | | 0 Yes | 0 No | |
| Which profession: | | |  | |  | | | |  |  | |
| 1b. Have you grown up around animals: | | | | |  | | | | 0 Yes | 0 No | |
| What sort of animals: | | | | |  | | | |  |  | |
| 1c. Do you have pets: | | |  | |  | | | | 0 Yes | 0 No | |
| What kind of pets: | | |  | |  | | | |  |  | |
| 2a. Have you ever travelled to Africa? | | | | | |  | | | 0 Yes | 0 No | |
| How often? | | | |  | |  | | |  |  | |
| Which countries? | | | |  | |  | | |  |  | |
| 2b. Have you ever travelled to Asia? | | | | | |  | | | 0 Yes | 0 No | |
| How often? | | | |  | |  | | |  |  | |
| Which countries? | | | |  | |  | | |  |  | |
| 2c. Have you ever been on a safari to see wild animals? | | | | | | | | | 0 Yes | 0 No | |
| How often? | | | |  | |  | | |  |  | |
| Which countries? | | | |  | |  | | |  |  | |
| 2d. Have you ever undertaken other tourist activities which involved wild animals? | | | | | | | | | 0 Yes | 0 No | |
| What type of activities? | | | | | |  | | |  |  | |
| How often? | | | | | |  | | |  |  | |
| In which country/countries? | | | | | |  | | |  |  | |
| 3a. Do you ever visit the zoo? | | | | | |  | | | 0 Yes | 0 No | |
| How often? | | | |  | |  | | |  |  | |
| Which zoo(s)? | | | |  | |  | | |  |  | |
| 3b. Do you ever watch animal or nature related documentaries? | | | | | | | | | 0 Yes | 0 No | |
| How often? | | | |  | |  | | |  |  | |
| Which documentary/documentaries? | | | |  | | | |  |  |  | |
| Which television channel? | | | |  | |  | | |  |  | |
| 3c. Do you ever read animal or nature related magazines? | | | | | | | | | 0 Yes | 0 No | |
| How often? | | | |  | |  | | |  |  | |
| Which magazine(s)? | | | |  | |  | | |  |  | |

***<PRESENT PHOTOGRAPH> I would now like to ask you to take a look at this photograph***

| 4. Have you ever seen this animal? | | | | | | 0 Yes | | 0 No |
| --- | --- | --- | --- | --- | --- | --- | --- | --- |
| Where have you seen it? | | |  |  | |  | |  |
| Can you tell me the name of this animal? | | | |  | |  | |  |
| 5a. Would you like to be photographed with this animal? | | | | 0 Yes | | 0 Maybe | | 0 No |
| 5b. Would you like to walk with this animal? | | | | 0 Yes | | 0 Maybe | | 0 No |
| 5c. Would you like to pet this animal? | | | | 0 Yes | | 0 Maybe | | 0 No |
| 5d. Do you think this animal is a nice pet? | | | | 0 Yes | | 0 Maybe | | 0 No |
| 6a. How suitable or unsuitable do you think this animal is to be photographed with tourists: | | | | | | | | |
| 0 Very suitable | 0 Suitable | | 0 Not suitable but also not unsuitable | | | 0 Unsuitable | | 0 Very unsuitable |
| 6b. How suitable or unsuitable do you think this animal is to walk with tourists: | | | | | | | | |
| 0 Very suitable | 0 Suitable | | 0 Not suitable but also not unsuitable | | | 0 Unsuitable | | 0 Very unsuitable |
| 6c. How suitable or unsuitable do you think this animals is to be petted by tourists: | | | | | | | | |
| 0 Very suitable | 0 Suitable | | 0 Not suitable but also not unsuitable | | | 0 Unsuitable | | 0 Very unsuitable |
| 6d. How suitable or unsuitable do you think this animal is to be kept by people as a pet: | | | | | | | | |
| 0 Very suitable | 0 Suitable | | 0 Not suitable but also not unsuitable | | | 0 Unsuitable | | 0 Very unsuitable |
| 7. Do you think this animal is: | | | | | | | | |
| 0 Very sweet | | 0 Sweet | 0 Not sweet but also not dangerous | | 0 Dangerous | | 0 Very dangerous | |
| 8. Do you think in the wild this animal is: | | | | | | | | |
| 0 Not endangered | | | 0 Endangered | | | 0 Very endangered | | |

| **Foto gebruikt tijdens het interview** | | | | | | | | | | |
| --- | --- | --- | --- | --- | --- | --- | --- | --- | --- | --- |
| **0 Caracal** | | **0 Cheeta** | | | | | **0 Leeuw** | | |  |
| **0 Controle** | | **0 Lopen** | | | | | **0 Aaien** | | | **0 Wild** |
| Datum: | | Locatie: | | | | | Naam interviewer: | | | |
| Geslacht respondent: | | | 0 Man | | 0 Vrouw | | |  |  | |
| Wat is uw leeftijd? | | | | |  | | |  |  | |
| Wat is uw hoogst afgeronde opleiding? | | | | | | | | 0 VMBO | 0 HAVO | |
| 0 VWO | 0 MBO | | 0 HBO | | 0 WO | | | 0 Anders: | | |
| In welk dorp/plaats/stad woont u: | | | | | | | |  |  | |
| 1a. Heeft u een beroep waarin u contact heeft  Met dieren: | | | | | | | | 0 Ja | 0 Nee | |
| Welk beroep: | | |  | |  | | |  |  | |
| 1b. Bent u opgegroeid met dieren: | | | | |  | | | 0 Ja | 0 Nee | |
| Wat voor soort dieren: | | | | |  | | |  |  | |
| 1c. Heeft u huisdieren: | | |  | |  | | | 0 Ja | 0 Nee | |
| Wat voor soort huisdieren: | | |  | |  | | |  |  | |
| 2a. Bent u wel eens in Afrika geweest? | | | | | |  | | 0 Ja | 0 Nee | |
| Hoe vaak? | | | |  | |  | |  |  | |
| Welke landen? | | | |  | |  | |  |  | |
| 2b. Bent u wel eens naar Azië geweest? | | | | | |  | | 0 Ja | 0 Nee | |
| Hoe vaak? | | | |  | |  | |  |  | |
| Welke landen? | | | |  | |  | |  |  | |
| 2c. Bent u wel eens op safari geweest om wilde dieren te zien? | | | | | | | | 0 Ja | 0 Nee | |
| Hoe vaak? | | | |  | |  | |  |  | |
| Welke landen? | | | |  | |  | |  |  | |
| 2d. Heeft u wel eens een andere toeristische activiteit met wilde dieren ondernomen? | | | | | | | | 0 Ja | 0 Nee | |
| Wat voor soort activiteiten? | | | | | |  | |  |  | |
| Hoe vaak? | | | | | |  | |  |  | |
| In welk land/landen? | | | | | |  | |  |  | |
| 3a. Gaat u wel eens naar de dierentuin? | | | | | |  | | 0 Ja | 0 Nee | |
| Hoe vaak? | | | |  | |  | |  |  | |
| Welke dierentuin(en)? | | | |  | |  | |  |  | |
| 3b. Kijkt u wel eens naar dier of natuur gerelateerde documentaires? | | | | | | | | 0 Ja | 0 Nee | |
| Hoe vaak? | | | |  | |  | |  |  | |
| Welke documentaire/documentaires? | | | |  | |  | |  |  | |
| Welke televisiezender? | | | |  | |  | |  |  | |
| 3c. Leest u wel eens dier of natuur gerelateerde magazines? | | | | | | | | 0 Ja | 0 Nee | |
| Hoe vaak? | | | |  | |  | |  |  | |
| Welke magazine(s)? | | | |  | |  | |  |  | |

***<PRESENTEER DE FOTO> Ik wil u nu vragen naar deze foto te kijken***

| 4. Heeft u dit dier ooit gezien? | | | | | | 0 Ja | | 0 Nee |
| --- | --- | --- | --- | --- | --- | --- | --- | --- |
| Waar heeft u het gezien? | | |  |  | |  | |  |
| Kunt u mij de naam van dit dier vertellen? | | | |  | |  | |  |
| 5a. Zou u met dit dier op de foto willen? | | | | 0 Ja | | 0 Misschien | | 0 Nee |
| 5b. Zou u met dit dier willen wandelen? | | | | 0 Ja | | 0 Misschien | | 0 Nee |
| 5c. Zou u dit dier willen aaien? | | | | 0 Ja | | 0 Misschien | | 0 Nee |
| 5d. Denkt u dat dit dier een leuk huisdier is? | | | | 0 Ja | | 0 Misschien | | 0 Nee |
| 6a. Hoe geschikt of ingeschikt denkt u dat dit dier is om met toeristen op de foto te gaan: | | | | | | | | |
| 0 Heel geschikt | 0 Geschikt | | 0 Niet geschikt maar ook niet ongeschikt | | | 0 Ongeschikt | | 0 Heel ongeschikt |
| 6b. Hoe geschikt of ongeschikt denkt u dat dit dier is om met toeristen te wandelen: | | | | | | | | |
| 0 Heel geschikt | 0 Geschikt | | 0 Niet geschikt maar ook niet ongeschikt | | | 0 Ongeschikt | | 0 Heel ongeschikt |
| 6c. Hoe geschikt of ongeschikt denkt u dat dit dier is om door toeristen geaaid te worden: | | | | | | | | |
| 0 Heel geschikt | 0 Geschikt | | 0 Niet geschikt maar ook niet ongeschikt | | | 0 Ongeschikt | | 0 Heel ongeschikt |
| 6d. Hoe geschikt of ongeschikt denkt u dat dit dier is om door mensen als huisdier gehouden te worden: | | | | | | | | |
| 0 Heel geschikt | 0 Geschikt | | 0 Niet geschikt maar ook niet ongeschikt | | | 0 Ongeschikt | | 0 Heel ongeschikt |
| 7. Denkt u dat dit dier: | | | | | | | | |
| 0 Heel life is | | 0 Lief is | 0 Niet lief maar ook niet gevaarlijk is | | 0 Gevaarlijk is | | 0 Heel gevaarlijk is | |
| 8. Denkt u dat dit dier in het wild: | | | | | | | | |
| 0 Niet met uitsterven bedreigd is | | | 0 Met uitsterven bedreigd is | | | 0 Heel erg met uitsterven bedreigd is | | |
